# Mechanistic Connections between Endoplasmic Reticulum (ER) Redox Control and Mitochondrial Metabolism

**DOI:** 10.3390/cells8091071

**Published:** 2019-09-12

**Authors:** Yuxiang Fan, Thomas Simmen

**Affiliations:** 1 Department of Cell Biology, Faculty of Medicine and Dentistry, University of Alberta, Edmonton, AB T6G2H7, Canada; yuxiang@ualberta.ca; 2 Department of Neurosurgery, the First Hospital of Jilin University, Changchun 130021, China

**Keywords:** endoplasmic reticulum, mitochondria, redox, chaperones, autophagy, ER-phagy

## Abstract

The past decade has seen the emergence of endoplasmic reticulum (ER) chaperones as key determinants of contact formation between mitochondria and the ER on the mitochondria-associated membrane (MAM). Despite the known roles of ER–mitochondria tethering factors like PACS-2 and mitofusin-2, it is not yet entirely clear how they mechanistically interact with the ER environment to determine mitochondrial metabolism. In this article, we review the mechanisms used to communicate ER redox and folding conditions to the mitochondria, presumably with the goal of controlling mitochondrial metabolism at the Krebs cycle and at the electron transport chain, leading to oxidative phosphorylation (OXPHOS). To achieve this goal, redox nanodomains in the ER and the interorganellar cleft influence the activities of ER chaperones and Ca^2+^-handling proteins to signal to mitochondria. This mechanism, based on ER chaperones like calnexin and ER oxidoreductases like Ero1α, controls reactive oxygen production within the ER, which can chemically modify the proteins controlling ER–mitochondria tethering, or mitochondrial membrane dynamics. It can also lead to the expression of apoptotic or metabolic transcription factors. The link between mitochondrial metabolism and ER homeostasis is evident from the specific functions of mitochondria–ER contact site (MERC)-localized Ire1 and PERK. These functions allow these two transmembrane proteins to act as mitochondria-preserving guardians, a function that is apparently unrelated to their functions in the unfolded protein response (UPR). In scenarios where ER stress cannot be resolved via the activation of mitochondrial OXPHOS, MAM-localized autophagosome formation acts to remove defective portions of the ER. ER chaperones such as calnexin are again critical regulators of this MERC readout.

## 1. Introduction

**Figure 1:**
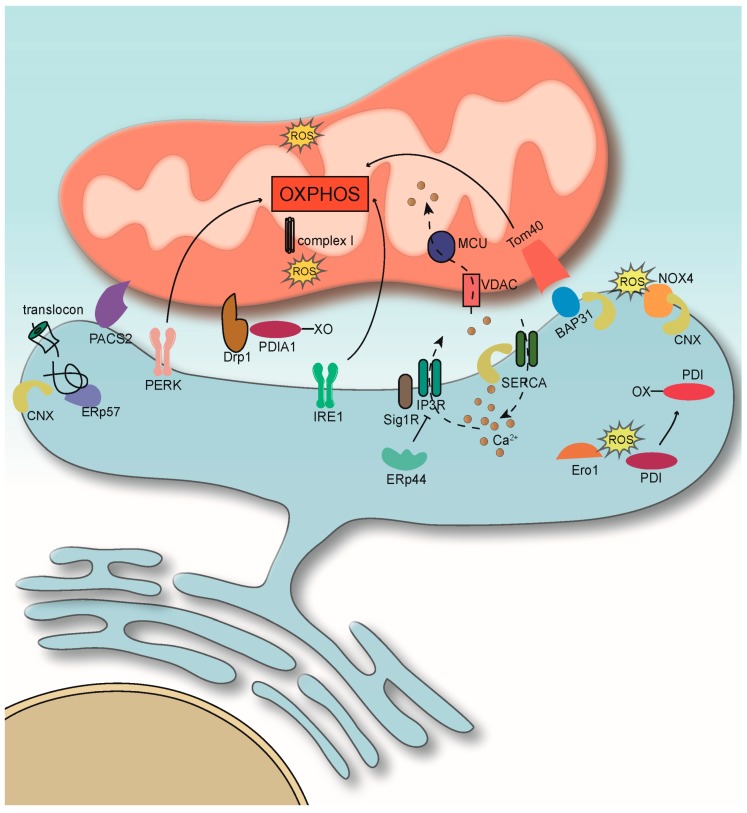
Endoplasmic reticulum (ER)–mitochondria interactions under homeostatic conditions. Unfolded protein response (UPR)-unrelated activities of Ire1 and PERK control mitochondrial metabolism. Ca^2+^ homeostatic flux from the ER to mitochondria maintains the Krebs cycle. ER chaperones keep this flux within normal settings. For details, see text.

Close to 10 years ago, we proposed a tight regulatory connection between oxidative protein folding within the endoplasmic reticulum (ER) and mitochondrial oxidative phosphorylation (OXPHOS) [1]. At the time, the unexpected discovery of ER chaperones and oxidoreductases in the proximity of mitochondria had prompted us to hypothesize that ER oxidative protein folding and OXPHOS entertain a special relationship that is based on mutual, bidirectional interaction. However, besides an enrichment of these folding enzymes on the mitochondria–ER contact sites (MERCs), we could not provide much evidence for such a hypothesis at the time. Some of the limited evidence included a role of substrate-restricted ER chaperones for OXPHOS [2]. For instance, sigma-1 receptors (Sig1Rs) stabilize inositol 1,4,5-trisphosphate receptors (IP_3_Rs) on MERCs, thus maintaining Ca^2+^ signaling from the ER to mitochondria [3]. Assisting this role is the oxidoreductase Ero1α, which is a component of the mitochondria-associated membrane (MAM) proteome under resting conditions [4] and activates IP_3_Rs to facilitate mitochondrial permeability transitions [5]. Today, a much wider array of proteins is known to control mitochondrial and cellular metabolism from the MERCs (Figure 1).

**Figure 2:**
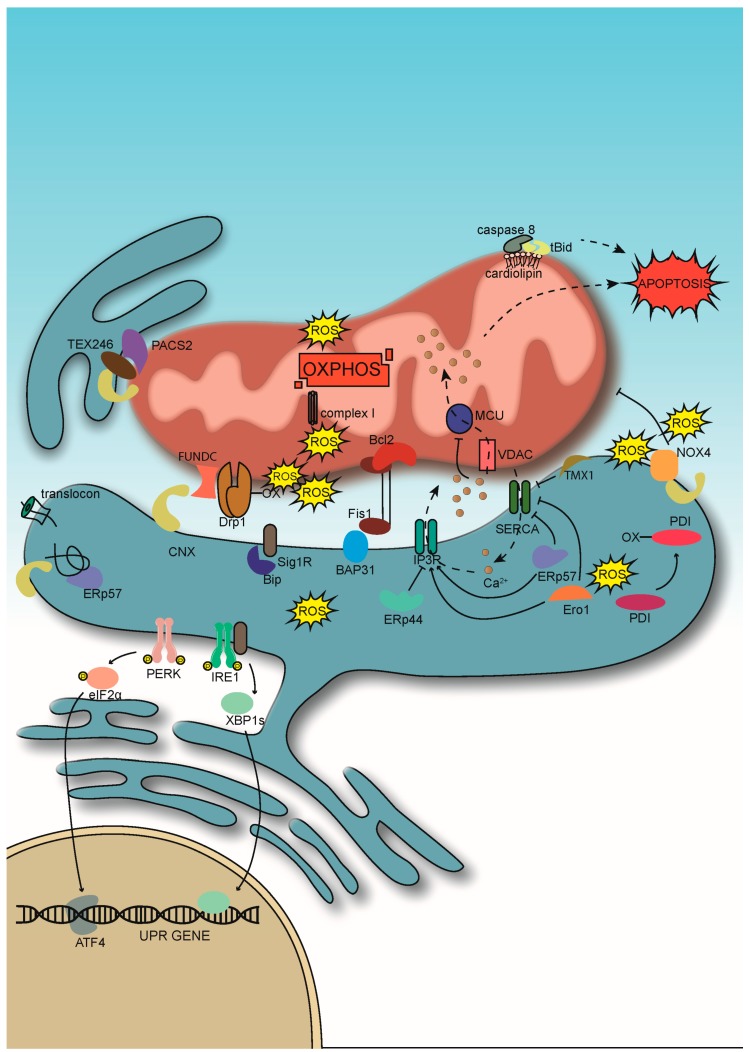
ER–mitochondria interactions under stress conditions. Basal reactive oxygen species (ROS) production increases, leading to a transfer of Ca^2+^ from the ER to mitochondria, initially boosting oxidative phosphorylation (OXPHOS), but ultimately triggering apoptosis and autophagy. ER chaperones and oxidoreductases alter their homeostatic interaction patterns and associate with pro-apoptotic and pro-autophagic machineries. For details, see text.

However, the induction of ER stress from the accumulation of unfolded proteins reveals a much broader functional link between chaperones and mitochondrial metabolism (Figure 2). This cellular condition results in increased production of reactive oxygen species (ROS) and activates a cellular signaling mechanism that counteracts the accumulation of unfolded proteins, called the “unfolded protein response” (UPR). The UPR has three main pathways based on the ER-localized sensor proteins inositol-requiring protein 1α (Ire1α), PKR-like ER kinase (PERK), and activating transcription factor 6 (ATF6), normally kept inactive via immunoglobulin-binding protein (BiP/GRP78) [6]. These proteins induce the production of chaperones while repressing the production of secretory proteins. However, the UPR is not restricted to controlling the folding environment within the ER. Early on, upon the discovery of ER-localized caspases, it had been reported that an extended UPR can also trigger apoptosis [7]. Such an ER-specific cell death pathway requires pro-apoptotic Bcl2 family proteins [8], whose activity is controlled by the BH3-only proteins Puma and Bim [9,10]. Another key player in ER-derived apoptosis is the transmembrane protein BAP31 that has chaperone functions in both mammalian and yeast model systems [11,12], but also associates with caspases and the lectin chaperone calnexin [13]. Upon cell stress, BAP31 is able to trigger apoptotic Ca^2+^ transfer to mitochondria [14]. This latter function requires the formation of a complex of BAP31 with Bcl2 and the mitochondrial fission protein Fis1 [15]. In contrast, under resting conditions, BAP31 interacts with Tom40 to stimulate mitochondrial OXPHOS at complex I [16]. The BAP31 paradigm shows nicely how extended ER stress acts as a decisive determinant between the roles of MERC protein complexes in metabolism or apoptosis. A key regulatory role in these mechanisms may fall onto Bax inhibitor 1 (BI-1), a MAM-localized interactor of Bax, since it can control ER ROS production [17,18], which could also determine the oxidation of mitochondrial Ca^2+^-handling proteins such as the mitochondrial Ca^2+^ uniporter (MCU) [19].

In addition to apoptosis and OXPHOS, ER chaperone dysfunction also influences mitochondrial membrane dynamics. At early stages, the accumulation of unfolded proteins within the ER leads to stress-induced hyperfusion of mitochondria [20]. Given that hyperfused mitochondria correlate with increased OXPHOS [21], this finding suggests that ER stress initially activates mitochondria. Indeed, such observations have been made [22,23]. Very recently, the maintenance of sarco/endoplasmic reticulum Ca^2+^ ATPase (SERCA) activity has been described as necessary for this activity [24]. Subsequently, increased ATP import via the ER-localized ATP transporter SLC35B1/AXER [25] aims to resolve ER stress. Tightened ER–mitochondria contact sites mediate this ATP transport, but this metabolic response is later transformed into an apoptotic one, where the release of cytochrome c coincides with a disruption of mitochondrial cristae [26]. More and more protein complexes containing ER chaperones and oxidoreductases that control these decision-making processes at MERCs are emerging. 

Lipid metabolism is the originally described function of the MAM [27,28]. This function is interestingly also connected to the UPR: Lipid imbalance can cause the activation of the UPR sensor proteins in the absence of unfolded proteins [29], but it is likely that this is not a bona fide ER stress readout. Moreover, cholesterol saturation of ER membranes, monitored by the sterol-responsive element binding protein (SREBP) [30], also triggers the UPR [31]. Inversely, a UPR induction leads to the activation of SREBP, followed by increased production of cholesterol [32]. This activation requires the action of ER-localized caspase-2 [33]. Under this condition and similar to the classic induction of a UPR, MERCs increase as well [34], but Ca^2+^ uptake to the ER decreases [35]. Together, these findings propose an avenue for how altered lipid production and handling could have a wider significance for ER homeostasis that depends on the extent and quality of MERC formation. Potentially, these functional links could also explain the induction of ER stress upon knockout of MERC tethering factors such as mitofusin-2 [36] and PACS-2 [37].

As expected from a central role of ER protein folding in MERC maintenance and the control of mitochondrial apoptosis and metabolism, numerous ER chaperones and oxidoreductases that control some or all of these mechanisms have been identified. They will be the topic of this review. Moreover, we will attempt to identify unifying characteristics and mechanisms that allow MERCs to sense stress in the ER and to communicate such conditions over to mitochondria. Changes in ER redox conditions and accumulations of ROS that chemically modify MAM proteins are likely key to this crosstalk.

## 2. Sigma-1 Receptor (Sig1R) Controls a Subset of Contact Site Regulators

The non-opioid Sig1R is an ER chaperone with one transmembrane domain and a luminal C-terminus [38,39]. Sig1R does not show sequence homology to other mammalian proteins, but shows a high degree of homology to the Erg2 yeast Δ8/Δ7-sterol isomerase [40,41]. The receptor localizes to MERCs, where it interacts with and stabilizes IP_3_R3s unless bound to BiP/GRP78. This interaction ultimately allows the ER to increase Ca^2+^ flux towards mitochondria [3]. While under ER stress, Sig1R can also stabilize the UPR sensor protein Ire1 at ER–mitochondria contacts and promote its RNase activity [42]. This suggests that Sig1R is not limited to chaperoning specific substrates, but also has a homeostasis-maintaining role for the ER. This role is apparently lost upon prolonged ER stress of more than 30 mins or upon agonist binding, when Sig1R relocates from MERCs to other domains of the ER and the plasma membrane, where it interacts with ion channels [43]. These interactions frequently result in the inactivation of K^+^ [44] and Ca^2+^ channels [45]. In the case of ER-localized Sig1R agonist binding, IP_3_R3 activity increases [46]. Since these ion channels provide mitochondria with the Ca^2+^ needed for the dehydrogenases of the Krebs cycle, this subsequently activates mitochondrial OXPHOS and local ROS production at complex I [47]. Accordingly, upon depletion or knockout of Sig1R, mitochondrial ATP production decreases, leading to overall oxidative stress [48]. As a further consequence, Sig1R knockout interferes with the activation of the Nrf2 transcription factor [49]. These findings strongly indicate that Sig1R activity maintains physiological Ca^2+^ flux between the ER and mitochondria to maintain cellular energy levels. At the moment, the mechanistic basis for this function of Sig1R is not fully elucidated, and it is also not clear whether IP_3_R3 is solely responsible for it. Whether and how the Sig1R reacts to oxidative stress are currently not known.

This relationship between oxidative stress and the Sig1R is also relevant in disease. For instance, in the context of superoxide dismutase-linked, inherited juvenile amyotrophic lateral sclerosis (ALS), Sig1R mutations cause MERC and mitochondrial OXPHOS deficiencies that result in disrupted ER–mitochondria Ca^2+^ flux [50]. The same is observed in distal hereditary motor neuropathies [51]. Moreover, the Sig1R mouse knockout model exhibits late onset retinal degeneration [52] associated with endogenous oxidative stress [53] and Nrf2 interference [54]. The information about the physiological role of Sig1R therefore corroborates a role as a guardian of mitochondrial functions and a preventor of oxidative stress during homeostatic conditions.

## 3. Calnexin and BAP31 Form a Chaperone-Based Nexus to Control Multiple MERC Functions

The ER Ca^2+^-binding chaperone calnexin binds monoglucosylated peptides upon their entry through the translocon and the trimming of their sugar structure by glucosidases I and II within the ER [55]. This role is especially important upon ER stress, when the association of calnexin with ERp57 increases [56], thus focusing its function on chaperoning and folding. However, calnexin is multifunctional and a significant portion of calnexin also localizes on ER–mitochondria contact sites [57], where it is one of very few reliably detected marker proteins [58,59]. Calnexin uses DHHC6-mediated palmitoylation of two cysteines close to its transmembrane domain to target to MERCs [60]. At least three functions that associate calnexin with mitochondria are currently known. 

Close to mitochondria, calnexin binds to SERCA [56]. Given the significantly lower Ca^2+^ uptake to the ER in the absence of calnexin, this interaction likely acts as an activation. It is currently not known whether the calnexin-dependent loading of the ER with Ca^2+^ could influence mitochondrial metabolism. An important binding partner of calnexin on MERCs is BAP31, which can interact with mitochondrial Tom40 [16]. BAP31 also interacts with mitochondrial NADH:ubiquinone oxidoreductase (mitochondrial complex I) core subunit 4 (NDUFS4), a known component of the MAM proteome [16]. In the absence of BAP31, mitochondrial OXPHOS is repressed, but it is not clear whether the binding to Tom40 is required for this function, since the MERC targeting mechanism of BAP31 is currently unknown.

Upon apoptosis induction or oxidative stress, calnexin moves away from MERCs and dissociates from BAP31 [61]. Under this condition, BAP31 increases its interaction with mitochondria by forming a complex with procaspase-8 and Fis1 [15]. This eventually triggers caspase-mediated cleavage of BAP31 [14]. Therefore, the BAP31–calnexin complex formed under non-stressed conditions presumably acts to maintain ER–mitochondria contacts. A third protein interacting with calnexin in the proximity of mitochondria is FUN14 domain-containing protein 1 (FUNDC1), which acts as an mitophagy receptor under hypoxia [62]. Similar to the interaction of calnexin with BAP31, this protein complex also dissolves under stress conditions, thus making FUNDC1 available for interaction with Drp1, which subsequently promotes mitophagy [63]. Therefore, overall, calnexin localizing to MERCs promotes homeostasis, a statement also supported by the recently discovered interaction of calnexin with acyl-CoA:diacylglycerol acyltransferase (DGAT2), another MAM marker protein that cooperates with calnexin in triacylglycerol metabolism [64]. The role of calnexin for cellular lipid homeostasis may extend into cholesterol, since calnexin can also bind to ATP-binding cassette transporter A1 (ABCA1), for which it mediates stability. In the absence of calnexin, ABCA1 is degraded, thus increasing cholesterol within the ER [65]. Since cholesterol is an integral and essential component of MAMs, this finding suggests that calnexin absence could increase contact formation between the ER and mitochondria, which could alter ATP and ROS output from mitochondria.

The multifunctionality of calnexin extends into the control of oxidative stress. At the ER, one of its binding partners is NADPH oxidase 4 (Nox4), whose ROS production is under the control of calnexin, albeit not involving its lectin property [66]. Together with Ero1α (see below), Nox4 is responsible for ROS output during ER stress [67]. While it is currently unclear whether Nox4 directly uses ROS to signal towards mitochondria, this enzyme-generated oxidative stress keeps glycolysis active via maintenance of Hif1α protein levels [68]. At the same time, high levels of Nox4 activity can coincide with repressed mitochondrial activity in a melanoma model, suggesting that calnexin could use Nox4-mediated ROS production to generally repress mitochondria [69]. This could be based on redox modifications of mitochondrial Ca^2+^-handling proteins [19] or could impact the ER–mitochondria redox crosstalk [70]. Via these functional links, calnexin might also control some types of mitochondrial apoptosis responses [61,71]. Together, calnexin appears to act mostly during homeostatic conditions, as indicated by its inhibitory actions on BAP31 and FUNDC1-mediated apoptosis and mitophagy. Via the maintenance of Nox4 ROS output, calnexin could also use redox signals to shift the cellular metabolic equilibrium from OXPHOS towards glycolysis.

In addition to calnexin’s control of MERC functions, further connections between ER protein folding and lipids depend on MERCs. For instance, MERC-localized BiP/GRP78 is critical for the folding of steroidogenic acute regulatory protein (StAR), which imports cholesterol into mitochondria, suggesting that the ER folding machinery controls ER–mitochondria contact formation also via the production and maintenance of MERC lipid transfer proteins [72]. Another aspect is the presence of a poorly understood platform of phosphatidic acid (PA) transfer from the ER to mitochondria on MERCs. This lipid serves as the precursor for mitochondrial cardiolipin [73] that is important for the assembly of mitochondrial OXPHOS supercomplexes [74], and fission-promoting Drp1 oligomers [75,76]. However, upon the accumulation of unfolded proteins within the ER and its associated oxidative stress, cardiolipin becomes oxidized [77], which subsequently causes the assembly of a pro-apoptotic protein complex containing Bid and Bax on the mitochondrial membrane. This then initiates apoptosis [78,79]. It is likely that additional functional connections exist between ER protein folding and ER lipid composition. These may also explain the induction of ER stress-like responses upon interference with lipid homeostasis. Future research will have to investigate these connections between ER chaperones, lipids, and oxidative stress further.

## 4. MERC Redox Control by Protein Disulfide Isomerase (PDI) Family Proteins and the Ero1 Oxidoreductase 

Recent progress indicates that ER–mitochondria contacts are important sites of ROS flux and transfer [80]. This suggests that ER redox-controlling enzymes determine when and how such a redox crosstalk occurs. The currently best-characterized function of ROS at MERCs is the control of ER–mitochondria Ca^2+^ signaling. For instance, IP_3_Rs are targets of ROS that oxidize distinct cysteines found in both the luminal and cytosolic portions of IP_3_Rs. ROS cause cysteine sulfenylation that can result in IP_3_R potentiation [81]. In contrast, both luminal and cytosolic SERCA oxidation tend to inactivate this Ca^2+^ pump [82,83]. ER oxidoreductases that act within the ER and on its cytosolic face are prominent regulators of these redox modifications. In this latter location, their function could depend on the formation of redox nanodomains based on the release of ROS from mitochondria under conditions of high cytosolic [Ca^2+^] that may spill over into the ER [70]. Given that the ER is itself a major source of ROS derived from oxidative protein folding [84] and Nox4 [67], such redox nanodomains could also originate at the ER.

Protein disulfide isomerase (PDI) proteins have one or more redox-active Cys–X–X–Cys motifs within thioredoxin-like domains [85,86]. Using these motifs and domains, PDI family proteins form and disrupt disulfide bonds to facilitate proteostasis together with myriads of chaperones [87]. A classic example is the PDI family member ERp57 that cooperates with calnexin and calreticulin to form disulfide bonds within mono-glucosylated, newly synthesized proteins such as the major histocompatibility complex class 1 (MHC class I) [88]. However, most PDI family members are multifunctional like ER chaperones, and some of them also control cellular metabolism. ERp57 is no exception. The main mechanism that PDI family proteins use to control cellular metabolism appears to be based on a control of Ca^2+^-based signaling [89]. ERp57 appears to oxidize multiple Ca^2+^-handling proteins, including SERCA2b [82] and STIM1 [90], with the goal to decrease ER Ca^2+^ uptake, albeit with currently unclear catalytic activity [91]. In addition to regulating the Ca^2+^ flux across ER and plasma membranes, ERp57 also participates in the modulation of Ca^2+^ homeostasis by maintaining MCU expression and, thus, mitochondrial Ca^2+^ uptake [92]. However, a mechanistic basis for this observation is currently unclear. Overall, ERp57 appears to increase mitochondrial Ca^2+^ retention at the expense of the ER. This could allow ERp57 to tweak the metabolic role of MERCs towards OXPHOS.

The thioredoxin luminal ER protein PDI is the founding member of this family of oxidoreductases. It is a critical component of ER oxidative protein folding that results in native disulfide bonds. In its most common enzymatic pathway, PDI receives an oxidizing equivalent from the oxidoreductase Ero1 to hand it over to newly synthesized proteins [93]. Potential links of PDI to cellular metabolism are suggested by its responsiveness to hypoxic insults [94]. At the moment, few such links between PDI and metabolism are known. However, a recent intriguing study found that the cytosolic moiety of PDIA1 reduces the cysteine 644 residue of the dynamin-related GTPase Drp1, a protein mediating mitochondria fission [95]. Via this activity, cytosolic PDIA1 acts as a reductase, thereby preventing Drp1 activation via sulfenylation and nitrosylation [96], two post-translational modifications that result in Drp1 oligomerization and activation. The same cytosolic moiety of PDI may also be able to trigger Bak oligomerization and, thus, apoptosis [97]. These unexpected functions of cytosolic PDI are exciting, since they provide the first mechanistic insight into how cytosolic redox nanodomains between the ER and mitochondria could modulate each other’s function. However, they also raise a lot of questions, for instance regarding the targeting of PDI to this unexpected locale. 

Interestingly, PDI itself is also subject to redox-mediated inactivation via S-nitrosylation during ER stress [98]. If occurring for an extended time, this inactivation can cripple mitochondrial function and result in cytochrome c release [99]. Another link between PDI activity and mitochondria has been revealed via the treatment with mitochondrial uncouplers that results in decreased levels of fumarate. This Krebs cycle intermediate normally binds covalently as succinate to PDI, leading to PDI inactivation and increased levels of ER stress. The exposure of cells to high levels of glucose increases this stress-generating modification, because, overall, it activates the production of Krebs cycle intermediates that can exit mitochondria and covalently modify PDI [100]. PDI therefore appears to act as an OXPHOS-sensing and maintaining enzyme. 

Connections between other PDI family proteins and metabolism are less well-characterized and understood. For instance, the PDI family member P5 that can also localize to mitochondria could maintain or even increase Krebs cycle activity under oxidative stress by sustaining the function of citrate synthase [101], a function that would ultimately suppress the release of cytochrome c and the generation of mitochondrial superoxide [102]. Functional connections also exist between additional PDI family members and SERCA. This ER Ca^2+^ pump shows activity levels that are dependent on the cellular and ER oxidation states [103]. For instance, TMX1 is a transmembrane PDI family protein that targets to the mitochondria-proximal portion of the ER [57,104]. Here, TMX1 interacts with SERCA2b, antagonizing calnexin, and inactivates this ER Ca^2+^ pump, resulting in the activation of mitochondrial OXPHOS similar to PDI [105].

The main function of the oxidoreductase Ero1α is to recharge PDI with oxidizing equivalents [106]. From this enzymatic activity, Ero1α produces ROS, but these typically neither accumulate within the ER nor do they significantly leak from the ER, even with massive over-expression of Ero1α [107]. The mechanism used by the ER to eliminate these excess ROS is to use them for further disulfide bond generation with the enzymes GPx7, GPx8, and peroxiredoxin 4 [108,109]. Of these, GPx8 localizes in a highly specific manner to MERCs, where it acts as a redox-dependent reducer of ER–mitochondria Ca^2+^ flux [110]. Like GPx8 and despite a role in ER oxidative protein folding thought to occur on the rough ER, Ero1α is highly enriched on MERCs [4]. Here, like PDI family proteins, Ero1α influences ER–mitochondria Ca^2+^ flux via multiple mechanisms, thus acting to control cellular metabolism. For instance, with regards to SERCA, Ero1α catalyzes the hyperoxidation of luminal cysteines within SERCA [111], a function potentially opposed by ERdj5. While Ero1α therefore inactivates SERCA, the PDI-related ERdj5 activates SERCA2b via the reduction of some of its cysteines. Thus, Ero1α and ERdj5 might have opposite significance for cellular metabolism [112]. In contrast to this role focusing on Ca^2+^ uptake, Ero1α can also oxidize and activate IP_3_R, thus increasing Ca^2+^ release from the ER, likely dependent on its localization to MERCs [4,113]. Opposing this function, ERp44 inhibits IP_3_R1-mediated Ca^2+^ release from the ER, but the significance of this finding for the cellular metabolism is currently unknown [114].

Overall, Ero1α therefore acts to decrease ER Ca^2+^. Like ERp57, Ero1α shifts the cellular Ca^2+^ equilibrium towards mitochondria, albeit via reduced SERCA pumping and increased IP_3_R release in this case. Inside mitochondria, MCU Ca^2+^-uptake activity requires Ero1α [113], further suggesting an OXPHOS-promoting role for this oxidoreductase. At the moment, we do not know whether this mitochondrial function originates from improper ER Ca^2+^ filling, ROS production, or is independent of either one. Moreover, it is unclear whether Ero1α concomitantly influences glycolysis.

## 5. Connections between UPR Signaling and Metabolism Mediated by PERK and Ire1

Oxidative protein folding and the import of Ca^2+^ into the ER are very energy-intensive mechanisms producing secretory proteins, thus making the ER one of the major sites of ATP consumption in the cell [115,116]. Cells answer to dysfunctional ER protein folding with the induction of a UPR [117]. This signaling mechanism relies on the interaction of ER transmembrane sensor proteins Ire1 and PERK that act as signaling emitters via the production of the Xbp1 transcription factor (Ire1) and the arrest of protein synthesis via the phosphorylation of eukaryotic initiation factor 2a (eIF2α; PERK). In contrast to these short-term responses, extended periods of ER stress equip both Ire1 and PERK with novel functions: While Ire1 becomes an mRNAse targeting multiple ER-associated mRNAs in a process termed “regulated Ire1-dependent mRNA decay” (RIDD) [118], PERK-mediated phosphorylation of eIF2α induces the production of the ATF4 transcription factor that activates downstream production of pro-apoptotic proteins [119].

Interestingly, novel insight was presented by Verfaillie and colleagues in 2012 when they showed that PERK localizes to ER–mitochondria contacts and promotes the formation of MERCs even in its UPR-dead form [77]. Via this MERC-promoting function, PERK acts to maintain mitochondrial apoptosis pathways. This elegant study tentatively established the first functional link between classic ER oxidative protein folding and the MAM, suggesting that the overall ER folding status and not just specialized chaperone–substrate interactions could determine MAM functions. Such a link was strengthened with the discovery that PERK must be expressed in cells for mitochondria to undergo elongation [20], as well as to ramp up their metabolism upon ER stress [120]. The two functions may be connected, since mitochondrial elongation is required to maintain mitochondrial bioenergetics [121]. These observations establish PERK as a key controller of OXPHOS, despite not localizing within mitochondria, but rather to the MERC.

However, the role of PERK in the maintenance of mitochondrial OXPHOS can also stem from transcriptional activities. For instance, the activation of PERK, leading to the phosphorylation of eIF2α, results in the subsequent production of the transcription factor ATF4 [122]. This sequence of events then acts to induce transcription of the supercomplex assembly factor (SCAF), a protein that anchors OXPHOS complexes on the mitochondrial inner membrane [120]. The role of PERK in controlling membrane contact sites (MCS) formed at the ER extends to connections with the plasma membrane. Here, dimerized PERK interacts with filamin A to rearrange the cortical actin cytoskeleton and increase the formation of MCS if cytosolic Ca^2+^ concentrations rise. This interaction then promotes the formation of ER–plasma membrane contacts [123].

More recently, Ire1α has also been detected on MERCs and has been implicated in the control of cellular metabolism. The induction of Ire1α signaling leads to reduced glycolysis [124]. Consistent with a role of Ire1α in cell metabolism, this ER stress-sensing protein has been detected as an interactor of IP_3_R3 that determines MERC structure, but also MERC Ca^2+^ signaling [125]. Unrelated to its transcriptional role in the UPR, Ire1α expression boosts mitochondrial OXPHOS [125]. At MERCs, Ire1α is also subject to ubiquitination by mitochondrial ubiquitin ligase (MITOL) [126], a covalent modification that prevents Ire1α-mediated mitochondrial apoptosis induction [127]. Interestingly, *Caenorhabditis elegans* Ire1 is subject to cytosolic sulfenylation derived from mitochondrial and ER sources, which induces a novel function of Ire1 that results in the activation of the MAP kinase pathway and the Nrf2 transcription factor [128]. In this paradigm, Nrf2 acts to induce antioxidant proteins and preserves mitochondrial metabolism [129]. This pathway is independent to the classic regulation of Nrf2 by Keap, and further highlights the central role of MERCs in the control of mitochondrial metabolism. Both PERK and Ire1 therefore exhibit functions that maintain mitochondrial metabolism at the expense of glycolysis. These roles in metabolism take advantage of the ER stress-monitoring properties of these two sensor proteins, but are otherwise not connected to their classic functions in the UPR.

## 6. MERC Tethering Factors: PACS-2 and Mitofusin-2

In 2005, the cytosolic phospho-furin acidic cluster sorting protein 2 (PACS-2) became the first known protein that promotes ER–mitochondria tethering [37]. Knockdown of this protein not only leads to reduced MERCs, as shown by a variety of laboratories and assays [130,131], but also induces a UPR [37]. This latter finding highlights the reciprocal nature of the UPR–MERC connection: Not only does oxidative protein folding influence MERC formation and signaling, but MERC disruption triggers a UPR. The reason for this association is currently unclear, but multiple factors could play a role, including the missorting of MERC proteins controlling ER homeostasis, the loss of mitochondrial ATP for oxidative protein folding, or the disruption of ROS and Ca^2+^ signaling between the ER and mitochondria. Further evidence for this connection comes from the increased MERC association of PACS-2 upon ER stress, suggesting that PACS-2 would act to increase ER–mitochondria contact formation under this condition [37]. Interestingly, this contact-boosting function could depend on Akt phosphorylation of PACS-2 on the MAM portion of the ER [132]. Although direct evidence for a role of PACS-2 in the balance between OXPHOS and glycolysis is currently missing, it is highly likely. Functions of PACS-2 unrelated to its role on MERCs also implicate it in the control of metabolism: It can block SIRT1-dependent p53 deacetylation [133], but it also allows for mTORC2 activation on MERCs via Akt [132], which are functions that could potentially explain the control of apoptosis by PACS-2 [134], as well as the protection from insulin resistance in PACS-2 knockout animals [130]. Whether altered ER homeostasis or oxidative stress play a role in these scenarios is currently unknown.

Mitochondrial membrane dynamics entertain a reciprocal relationship with MERCs. In addition, there are well-characterized links between mitofusins and metabolism, based on the roles of the mitofusins for mitochondria biogenesis and the production and maintenance of mitochondrial OXPHOS components and co-enzyme Q, but these are discussed elsewhere [135]. Instead, we will focus on the role of mitofusin-2 for MERCs in this review. While a role of mitofusin-2 for ER–mitochondria contacts is undisputed, it is currently unclear whether it controls the formation of MERC types [136] or MERCs as a whole [137]. Like in the case of PACS-2, a reciprocal link between MERCs and the UPR exists for mitofusin-2, whose absence via RNAi [138] and knockout also leads to ER stress [139,140]. Although it is not known whether the location of mitofusin-2 at MERCs increases upon a UPR, mitofusin-2 itself is a UPR target gene [141]. Mitofusin-2 promotes OXPHOS in proliferating cells, but it is not known whether this function is tied to its role in mitochondrial fusion or as a controller of MERCs [141].

Another tethering protein complex is based on the interaction between vesicle-associated membrane protein-associated protein B (VAPB) and the mitochondrial tyrosine phosphatase-interacting protein 51 (PTPIP51) [142]. While disruption of this complex compromises ER–mitochondria Ca^2+^ transfer and mitochondrial OXPHOS [143], it is unclear whether oxidative stress from the ER or mitochondria could influence this tether.

Together, the findings on PACS-2 and mitofusin-2 illustrate how the maintenance of MERCs or possibly their homeostatic structure promotes OXPHOS, albeit by currently unclear mechanisms. Of course, Ca^2+^-, lipid-, and ROS-based signaling is expected to cooperate to achieve this metabolic function of MERC tethering factors.

## 7. Links to Autophagy

ER stress and increases in ROS production at the MERC interface not only modulate the nature of the interorganellar interaction, but also activate autophagy with the goal to restore homeostasis. Therefore, MERCs have also been identified as an important point of origin for autophagosome membranes. This is an important functional link, since autophagosome formation is inhibited in the absence of the MERC tethering factors PACS-2 and mitofusin-2 [144]. Autophagy, including its best-characterized variant macroautophagy, serves to degrade or remodel stressed intracellular organelles [145]. The triggering of autophagy upon ER stress can selectively target the ER itself, a mechanism termed “ER-phagy” or “reticulophagy” [146,147,148] that depends on ATG2-mediated lipid transfer from the ER to the growing degradation container [149,150]. This subtype of autophagy is the main type of autophagy induced by ER stress and coincides with the induction of ATG8 in yeast [146]. In this model system, Atg39 and Atg40 have been identified as ER-specific autophagy receptors [151]. Atg40 is also a target of UPR-related transcriptional activation. This ER-specific autophagy receptor interacts with a COPII cargo adaptor complex formed by the yeast homologs of Sec23 and Sec24C (Lst1) on tubular ER junctions [152]. The importance of MERCs for autophagosome formation is reflected in a requirement of this subdomain of the ER for the autophagic elimination of mitochondria in yeast [153].

Mechanistically, ER stress-triggered autophagy involves persistent Ire1 and PERK oligomerization in mammalian cells. A consequence of this is the activation of c-Jun NH2-terminal kinase (JNK), a downstream target of Ire1 [154]. This kinase can then phosphorylate Bcl-2 to disrupt its inhibitory association with Beclin-1 [155]. Subsequently, the PERK downstream product ATF4 increases the production of ATG5, which then assembles with ATG12 and ATG16L to mediate lipidation of the ATG8 gene product [156]. In mammalian cells, several ATG8-related proteins exist in the form of multiple microtubule-associated proteins 1A/1B light chain 3 (LC3) and Gamma-aminobutyric acid receptor-associated proteins (GABARAPs) [157]. All of these are also targets of ATF4 [158]. ATG8-related proteins perform functions in autophagosome development and maturation, especially for initiation, elongation, and closure of autophagosomes, sometimes in an organelle-specific manner. Their connection to the UPR highlights the important role played by ER-phagy for the resolution of ER stress. During this condition, the ER has long been known to expand, a condition thought to increase the space of the ER for protein folding [159]. The expansion of the ER does require the activity of the UPR transcription factors Xbp1 [160] and ATF6 [161], which can both lead to increased production of phosphatidylcholine. Presumably, this increase in volume requires a temporary shutdown of ER-phagy and could depend on the ER-associated autophagy repressor Naf-1 [162].

In parallel with ATG8, ER stress leads to the induction of the LC3-interacting cell cycle progression gene 1 (CCPG1), a potential structural orthologue of Atg39 [151]. This autophagy receptor prevents the toxic accumulation of ER chaperones and folding intermediates during a UPR [163]. Following the associated increase in ER volume, after a UPR-inducing insult has been resolved, Sec62, a component of the translocon, uses its LC3-interactiong regions (LIR) in the cytosolic domain to further promote ER-phagy [164]. This function is critical for the recovery of ER homeostasis, and CCPG1 and Sec62 may therefore show temporal differences in their roles for ER-phagy [165].

Recent publications have led to the identification of novel ER-phagy regulatory proteins that make more direct connections to the ER protein folding machinery [148]. In particular, several receptors have been identified that contain LIRs and thus promote the specific degradation of ER membranes: For instance, family with sequence similarity 135B (FAM134B), a presumed functional counterpart of Atg40, alters ER membrane curvature with a reticulon homology domain [166]. A lack of FAM134B causes the ER to expand, while excess FAM134B activity fragments the ER via interaction with LC3 and GABARAP and subsequent targeting of ER portions to lysosomal degradation [167]. This mechanism uses calnexin as an autophagy co-receptor to target misfolded proteins for autophagy [168]. Similarly, but independent of FAM134B, the ER tubulation-promoting reticulon-3 binds via its LIR domains to LC3 and GABARAPs, inducing ER tubule fragmentation and their delivery to autophagosomes during starvation [169]. Since reticulons are important controllers of ER shape [170], this finding suggests that the structure of the ER is critical for the progression of ER-phagy, potentially via increasing the fluidity and allowing the remodeling of ER membranes necessary for their reassignment to autophagosomal structures. However, for this function of reticulons, LC3/GABARAP interaction is required, indicating that reticulons can moonlight as autophagy receptors. This is also the case with atlastin-3, an ER-associated GTPase that controls ER tubule fusion [171,172]. In this case, atlastin-3 binds to GABARAP, and this interaction is necessary to allow for ER-phagy upon starvation [173].

Out of all currently known ER autophagy receptors with an LIR, the testis-expressed protein 264 (TEX264) appears to show the strongest binding to LC3 and GABARAP [174]. The activity of this protein appears most important during nutrient stress, suggesting a role not directly connected to ER stress and redox [175]. However, TEX264 increasingly interacts with well-known MERC marker proteins such as calnexin and TMX1 under starvation conditions, suggesting that it may play a role for the selective removal of MAMs [175]. The autophagosome targeting of such portions of the ER may also be mediated by the Bcl2/adenovirus E1B 19kDa-interacting protein 3 (Bnip3), which interacts with LC3 and mediates ER-phagy and mitophagy dependent on its extent of targeting to either organelle [176].

Together, ER-phagy is intimately linked to the induction of the UPR, presumably with the aim to remove defective portions of the folding apparatus, but also to resolve stress-associated expansion of the ER. ER chaperones like calnexin assist in the formation of autophagosomes as co-receptors.

## 8. Conclusions and Perspectives

Increasing evidence confirms that ER oxidative protein folding acts as a “canary in the coalmine”, whose constant working is monitored by ER chaperones and oxidoreductases to adjust demands of energy and determine their sources. The redox events occurring at ER–mitochondria contact sites have wide significance for cellular physiology, but also for disease, since they determine the metabolic balance of energy generation. The best-known mechanism influencing this balance is based on ER–mitochondria Ca^2+^ transfer, but ER contacts with other organelles may emerge in the future as similarly important, especially since a few of the involved proteins also mediate other organellar contact sites.

However, disease-related mechanistic links are only emerging at this point. For instance, redox control by TMX1 at MERCs keeps ROS production from Nox4 in check, which can promote the growth of melanoma [69]. Another connection exists in the case of multiple sclerosis (MS). Here, oxidative stress leads to the activation of ER stress and the parallel induction of autophagy, detected via the induction of some autophagosome genes in patient samples, including ATG5 [177]. PACS-2 knockout protects from insulin resistance in the liver [131], but also in muscle [178]. Without any doubt, the future elucidation of mechanisms operating at MERCs will lead to a better understanding of how these intertwined organelles must work as a unit whose crosstalk must be tightly controlled. ER oxidative protein folding is a key mechanism that appears to control these functions of MAM proteins, suggesting that ER chaperones and oxidoreductases will emerge as important metabolic controllers in future research.
